# Genetic diversity and population structure of ALS‐resistant *Amaranthus hybridus* across Brazil's primary soybean‐growing regions

**DOI:** 10.1002/ps.8893

**Published:** 2025-05-13

**Authors:** Acácio Gonçalves Netto, Victor Hugo Vidal Ribeiro, Marcelo Nicolai, Ramiro Fernando Lopez Ovejero, Vanessa Francieli Vital Silva, Gilmar José Picoli Junior, Caio Brunharo

**Affiliations:** ^1^ Agro do Mato Soluções Agronômicas Santa Bárbara Brazil; ^2^ Department of Crop and Soil Science Oregon State University Corvallis Oregon USA; ^3^ Bayer Crop Science Brazil, Regulatory Science Sao Paulo Brazil; ^4^ Department of Plant Science Pennsylvania State University University Park Pennsylvania USA

**Keywords:** acetolactate synthase, independent evolution, smooth pigweed, target‐site resistance

## Abstract

**BACKGROUND:**

Resistance to acetolactate synthase (ALS)‐inhibiting herbicides has emerged in *Amaranthus hybridus* populations across Brazil's soybean‐growing regions. To gain insights into the evolutionary origins and spread of resistance, this study (1) investigated the ALS inhibitor resistance mechanisms in nine *A. hybridus* populations and (2) assessed their genetic diversity, structure, and relatedness.

**RESULTS:**

Resistance to the ALS inhibitor chlorimuron in *A. hybridus* was associated with two distinct target‐site mutations: Trp‐574‐Leu and Asp‐376‐Glu. Population genetics revealed low levels of genetic diversity (*H*_E_ = 0.00117 to 0.16019; π = 0.00126 to 0.17421) and inbreeding (*F*_IS_ = 0.0015 to 0.13157). Principal component analysis differentiated *A. hybridus* by geographical region, while ADMIXTURE analysis revealed population structure with evidence of admixture between genetic clusters in three groups of populations.

**CONCLUSION:**

The results suggest multiple local and independent evolutionary origins of resistance. The spread of resistance is primarily driven by local herbicide selection pressure and gene flow through seed dispersal. © 2025 The Author(s). *Pest Management Science* published by John Wiley & Sons Ltd on behalf of Society of Chemical Industry.

## Graphical abstract

We identified two distinct target‐site mutations, Asp‐376‐Glu and Trp‐574‐Leu, that confer resistance to acetolactate synthase inhibitors in *Amaranthus hybridus* populations across Brazil's soybean‐growing regions. These findings suggest multiple independent origins of resistance, with its spread primarily driven by local herbicide selection pressure and secondarily by seed‐mediated gene flow.

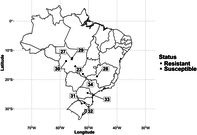

## 1 INTRODUCTION

Repeated and prolonged herbicide use has led to rapid weed adaptation across landscapes.^1^ Herbicide resistance in weeds is a classic example of adaptation to human‐mediated environmental change.^2^ Several weed species have adapted to thrive in these altered environments, including *Amaranthus hybridus* L. (smooth pigweed), which is a persistent weed in agricultural systems globally.^3^, ^4^, ^5^, ^6^

*Amaranthus hybridus* is native to North and South America^7^ and has been introduced to Europe, south‐central Asia, Africa, and Australia for its use as a leafy vegetable.^8^ It is a C_4_ summer annual species capable of producing over 250 000 seeds per plant.^9^ As a monoecious species, *A. hybridus* has both male and female flowers on the same plant and is considered primarily self‐pollinated due to the proximal arrangement of its flowers.^10^ This species is wind‐pollinated,^11^ and interspecific hybridization between *A. hybridus* and other *Amaranthus spp*. can occur under field conditions.^12^, ^13^, ^14^

In Brazil, *A. hybridus* is a difficult‐to‐control weed widely spread across soybean‐growing regions.^15^ If left uncontrolled, one *A. hybridus* plant m^−2^ can reduce soybean grain yield by 6.4%.^16^ Herbicides are the primary means of controlling *A. hybridus* in soybean production systems. However, overreliance on chemical control has resulted in the evolution of herbicide‐resistant populations. To date, *A. hybridus* populations have been identified in Brazil with resistance to acetolactate synthase (ALS)‐inhibiting herbicides^6^, ^15^, ^17^ and to the 5‐enolpyruvylshikimate‐3‐phosphate (EPSPS) inhibitor glyphosate.^17^, ^18^, ^19^ Some populations have evolved multiple resistance to both modes of action.^17^, ^20^

Herbicide resistance mechanisms in weeds can be either target‐site‐ or non‐target‐site‐based.^21^ Target‐site resistance (TSR) is conferred by amino acid substitutions that prevent effective inhibition of the herbicide target enzyme or other alterations that result in enhanced activity of the target‐site enzyme such as gene amplification (*i.e*., copy number variation) or overexpression.^22^ Non‐target site resistance (NTSR) mechanisms include enhanced metabolism, reduced absorption or translocation, or herbicide vacuolar sequestration.^23^ Despite the extensive reports of ALS inhibitor‐resistant *A. hybridus* populations in Brazil, the underlying resistance mechanisms have only recently been investigated. Two multiple‐resistant *A. hybridus* populations from southern Brazil were identified, with the Trp‐574‐Leu substitution in the *ALS* gene conferring cross‐resistance to imidazolinones and sulfonylureas.^17^ Furthermore, glyphosate‐resistant populations from southern Brazil, have the triple amino acid substitution Thr‐102‐Ile, Val‐103‐Ala, and Pro‐106‐Ser (TAP‐IVS)^17^, ^19^ and increased *EPSPS* copy number.^19^ The TAP‐IVS resistance mechanism was first reported in Argentina.^24^, ^25^ The introduction and rapid long‐distance spread of glyphosate‐resistant *A. hybridus* populations with the TAP‐IVS substitution in southern Brazil has been associated with agricultural machinery and seed crop contaminants,^26^ as well as pollen‐mediated gene flow over shorter distances.^19^

We previously conducted a 5‐year (2016–2020) herbicide resistance monitoring program across Brazil's primary soybean‐growing regions (the states of Bahia, Goiás, Maranhão, Minas Gerais, Mato Grosso, Mato Grosso do Sul, Pará, Piauí, Paraná, Rio Grande do Sul, Santa Catarina, São Paulo, and Tocantins), revealing widespread ALS resistance in *Amaranthus spp*.^15^ Of 226 *Amaranthus spp*. populations tested for resistance in this study, 26% were resistant to the ALS inhibitor chlorimuron. Nine of these populations were identified as *A. hybridus*, distributed across five states: Goiás, Minas Gerais, Mato Grosso, Paraná, and Rio Grande do Sul. The two most distant states, Rio Grande do Sul and Mato Grosso, are approximately 1930 km apart. This significant distance suggests that the spread of resistance could be due to multiple evolutionary origins or gene flow by seed. To test this hypothesis, our study aimed to (1) investigate the presence of nucleotide polymorphisms endowing ALS resistance in *A. hybridus* and (2) elucidate the evolutionary processes contributing to the spread of resistance by characterizing the genetic diversity, structure, and relatedness of ALS‐resistant *A. hybridus* populations across Brazil's primary soybean‐growing regions.

## 2 MATERIALS AND METHODS

### 2.1 Plant material

**Figure 1:**
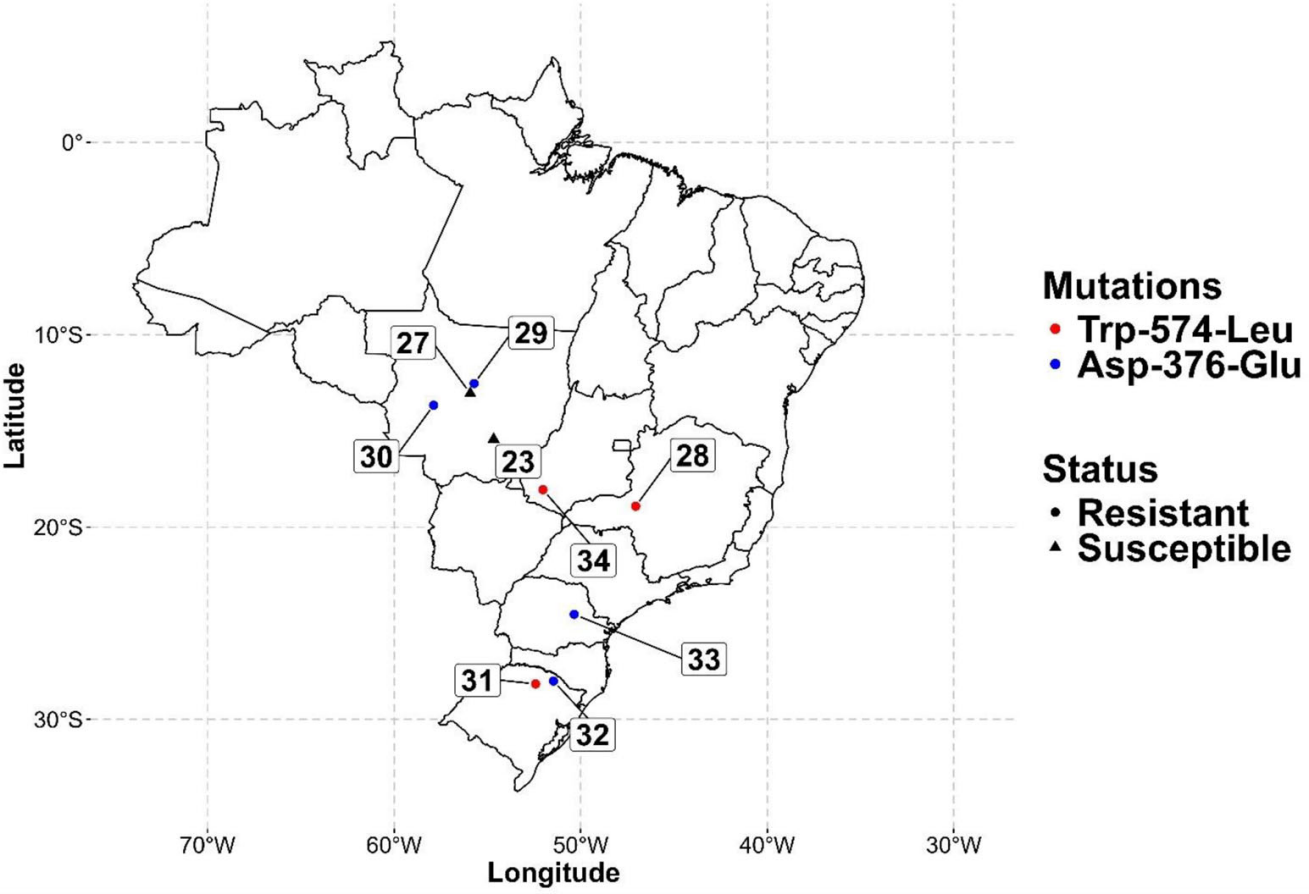
Geographic distribution of *Amaranthus hybridus* populations used in this study. Circles represent acetolactate synthase‐resistant populations; triangles represent acetolactate synthase‐susceptible populations. Blue circles indicate populations with the Asp‐376‐Glu mutation, and red circles indicate populations with Trp‐574‐Leu mutation.

Nine *A. hybridus* populations collected from soybean fields across five states in Brazil were used in this study (Fig. 1). These populations were collected between 2016 and 2020 as part of a herbicide resistance monitoring program for *Amaranthus spp*. across soybean production regions in Brazil. Dose–response studies were previously conducted on these populations with chlorimuron to determine the resistance levels.^15^ Chlorimuron was selected as a representative ALS inhibitor due to its widespread use in Brazil's soybean production systems. Seven ALS inhibitor‐resistant (28, 29, 30, 31, 32, 33 and 34) and two susceptible populations (23 and 27; Fig. 1) were included. Previous research showed that the GR_50_ (chlorimuron rate required to reduce biomass by 50%) for the resistant populations ranged from 37 to 100 g ai ha^−1^ of chlorimuron, compared to 8 to 16 g ai ha^−1^ for the susceptible populations.

### 2.2 DNA extraction and *ALS* sequencing

Acetolactate synthase resistance in weed species is typically target‐site based, resulting from amino acid substitutions at one of eight known positions (Ala‐122, Pro‐197, Ala‐205, Asp‐376, Arg‐377, Trp‐574, Ser‐653, and Gly‐654) within the five conserved *ALS* domains (A, B, C, D, and E).^27^ To test whether resistance to the ALS inhibitor chlorimuron in *A. hybridus* populations was caused by single nucleotide polymorphisms in the *ALS* gene, the entire gene region known to contain resistance‐conferring mutations was sequenced.

Plants selected for genotyping were part of a previously reported dose–response study.^15^ *Amaranthus hybridus* seeds were germinated in 2‐L plastic boxes filled with potting mix (pinus bark, peat, and vermiculite; 3:1; v/v). Seedlings at the 2‐true‐leaf stage were transplanted into 1‐L pots filled with potting mix. Experimental units consisted of a 1‐L pot containing three plants. Dose–response experiments were conducted to determine the resistance level by comparing resistant populations with two known susceptible populations.^15^ Additionally, 12 extra plants per treatment were included specifically for DNA collection and were sprayed alongside the dose–response experiments. Selection for genotyping was based on two criteria: (1) survival at a chlorimuron dose of 20 g ai ha^−1^ and (2) less than 80% visual injury (on a 0–100% scale, where 0 indicates no symptoms and 100% indicates complete control) 28 days after treatment. The only exception was population 34, for which resistance levels were not determined through dose–response experiments but rather by treatment with a discriminating rate of 20 g ai ha^−1^ of chlorimuron.^15^ Leaf tissue from susceptible genotypes was collected before herbicide treatment.

Approximately 50 mg of young leaf tissue was collected from eight plants of each *A. hybridus* population. DNA was extracted following Doyle and Doyle's (1987)^28^ protocol. The *ALS* gene region containing the eight known positions responsible for ALS resistance was amplified by PCR using the forward primer 5′‐ATGGCGTCCACTTCAACAAACC‐3′ and the reverse primer 5′‐CTAATAAGCCCTTCTTCCATCACCC‐3′.^29^ Thirty‐microliter reactions were prepared according to the standard protocol provided with Thermo Scientific Phusion polymerase (Waltham, MA, USA) and 20 ng of template genomic DNA. Reactions were cycled 32 times with the following three‐step protocol: 98 °C for 10 s, 62 °C for 20 s, and 72 °C for 90 s. PCR products were run on a 1% agarose gel to visualize fragment size. Purified PCR products were sequenced using both the aforementioned amplification primers and the following four sequencing primers: Seq_FP1 (5′‐AGTTTGTATTGCCACTTCTGGTCC‐3′), Seq_FP2 (5′‐GAAATCCTCGCCAATGGCTGAC‐3′), Seq_RP1 (5′‐GTCAGCCATTGGCGAGGATTTC‐3′), and Seq_RP2 (5′‐TGGACCAGAAGTGGCAATACAAAC‐3′).^29^

### 2.3 Population genomics of *Amaranthus hybridus* populations

To investigate the dynamics of independent evolution or gene flow among *A. hybridus* populations from different soybean‐growing regions in Brazil, *A. hybridus* populations were genotyped using a reduced representation DNA (RAD) sequencing approach.^30^, ^31^ The closely related *A. palmeri* was used as the outgroup population. Briefly, DNA was extracted from the same eight plants per population used in the targeted *ALS* sequencing experiment, and libraries were prepared by digesting DNA with *ApeKI* and *MseI* prior to barcoding, library quantification, and sequencing on an Illumina NextSeq 500 in 100 bp single read mode. Raw sequencing data were demultiplexed using the *process_radtags* program from Stacks (v2.64),^32^ followed by read alignment to the *A. hybridus* reference genome^33^ with *bwa mem* (v0.7.17),^34^ and sorting with *SAMtools* (v1.6).^35^ The *gstacks* program was used to build RAD loci and identify SNPs, and the *populations* program was applied for population filtering and quantification of population genetics statistics. SNPs were included only if they were present in at least 50% of the individuals within a population and across all populations (flags *‐r* and *‐p* in the *populations* program). Individuals with more than 50% missing genotypes were further filtered out using VCFtools.^36^ The final SNP dataset was used to explore the population genomics and generate summary statistics.

Population genomics estimates reflecting within‐ and between‐population variation were obtained. The expected heterozygosity (*H*_E_), nucleotide diversity (π), and inbreeding coefficient (*F*_IS_) were calculated with the *populations* module of Stacks. A Principal Component Analysis (PCA) was conducted to provide an initial assessment of the genetic relationship among populations. Separate analyses were performed with and without the outgroup species, with eigenvalues quantified using a custom R script,^37^ and plots were generated with ggplot2.^38^ Population structure was further analyzed using ADMIXTURE (v1.3.0),^39^ with ancestry values ranging from 2 to 10. Cross‐validation analysis, as recommended by Liu *et al*. (2020),^40^ was performed to determine the most likely value of *K*.

A phylogenetic tree was constructed to further dissect the genetic relationship among populations. Tree estimation was performed using the R package *ape* ^41^ with the *nj* function, following the calculation of pairwise genetic distances using the *adegenet* package.^42^ The phylogenetic tree was visualized with *ggtree* ^43^ and *treeio*.^44^ To investigate whether geographical distance among populations was associated with genetic distance, an isolation‐by‐distance test was conducted using *dist.genpop* function from *adegenet* package. The Mantel test, with 10 000 simulations, was performed within *adegenet*, and correlations were visualized with the *ggplot2* package.

## 3 RESULTS

### 3.1 *ALS* gene sequencing

The amplified *ALS* gene had 2010 bp covering the entire region known to contain resistance‐endowing mutations. Mutations at positions Trp‐574 and Asp‐376 were identified in the *A. hybridus* populations (Fig. 1). All sequenced individuals from resistant populations carried known ALS resistance‐endowing mutations. The Trp‐574‐Leu mutation (TGG‐TTG) was identified in populations 28, 31, and 34, while the Asp‐376‐Glu mutation (GAT‐GAG) was identified in populations 29, 30, 32, and 33. Populations 23 and 27 were susceptible to chlorimuron and had the wild‐type nucleotide sequence.

### 3.2 Population genomics of *Amaranthus hybridus* populations

Single‐read Illumina sequencing resulted in an average 2.42 ± 0.14 M (± SE) reads per sample, and our SNP calling routine identified 42 890 and 72 266 loci with and without *A. palmeri* as an outgroup, respectively, in downstream analyses. A total of 66 *A. hybridus* individuals, in addition to eight *A. palmeri* individuals, were retained in the population genetics analyses. Considerable variation was observed in the population genomics statistics of *A. hybridus* (Table 1). The expected heterozygosity (H_E_) varied from 0.00117 to 0.01033. Nucleotide diversity (π) followed a similar pattern to H_E_, ranging from 0.00126 to 0.01119. The inbreeding coefficients (F_IS_) ranged from −0.0015 to 0.02997. The outgroup had greater H_E_ (0.16019), π (0.17421), and F_IS_ (0.13157) values.

**Table 1:** Summary of population genetics statistics.^a^

Population	*H*_E_	π	*F*_IS_
23	0.00452 ± 0.0001	0.00489 ± 0.00011	0.00782 ± 0.00201
27	0.00304 ± 0.00007	0.00326 ± 0.00007	0.00844 ± 0.00158
28	0.00608 ± 0.0001	0.00659 ± 0.00011	0.0155 ± 0.0024
29	0.00731 ± 0.00013	0.00795 ± 0.00014	−0.0015 ± 0.00155
30	0.01033 ± 0.00013	0.01119 ± 0.00014	0.02997 ± 0.00209
31	0.00354 ± 0.00009	0.00381 ± 0.00009	0.00255 ± 0.00165
32	0.00473 ± 0.0001	0.00506 ± 0.00011	0.00938 ± 0.00129
33	0.00597 ± 0.00012	0.00641 ± 0.00013	0.01165 ± 0.00184
34	0.00117 ± 0.00004	0.00126 ± 0.00005	−0.00046 ± 0.00072
35	0.16019 ± 0.00042	0.17421 ± 0.00046	0.13157 ± 0.00329

^a^ Abbreviations: mean ± standard error; H_E_, expected heterozygosity; π, nucleotide diversity; F_IS_, inbreeding coefficient. Population 35 is an outgroup, *Amaranthus palmeri*. Populations 23 and 27 are ALS‐susceptible.

**Figure 2:**
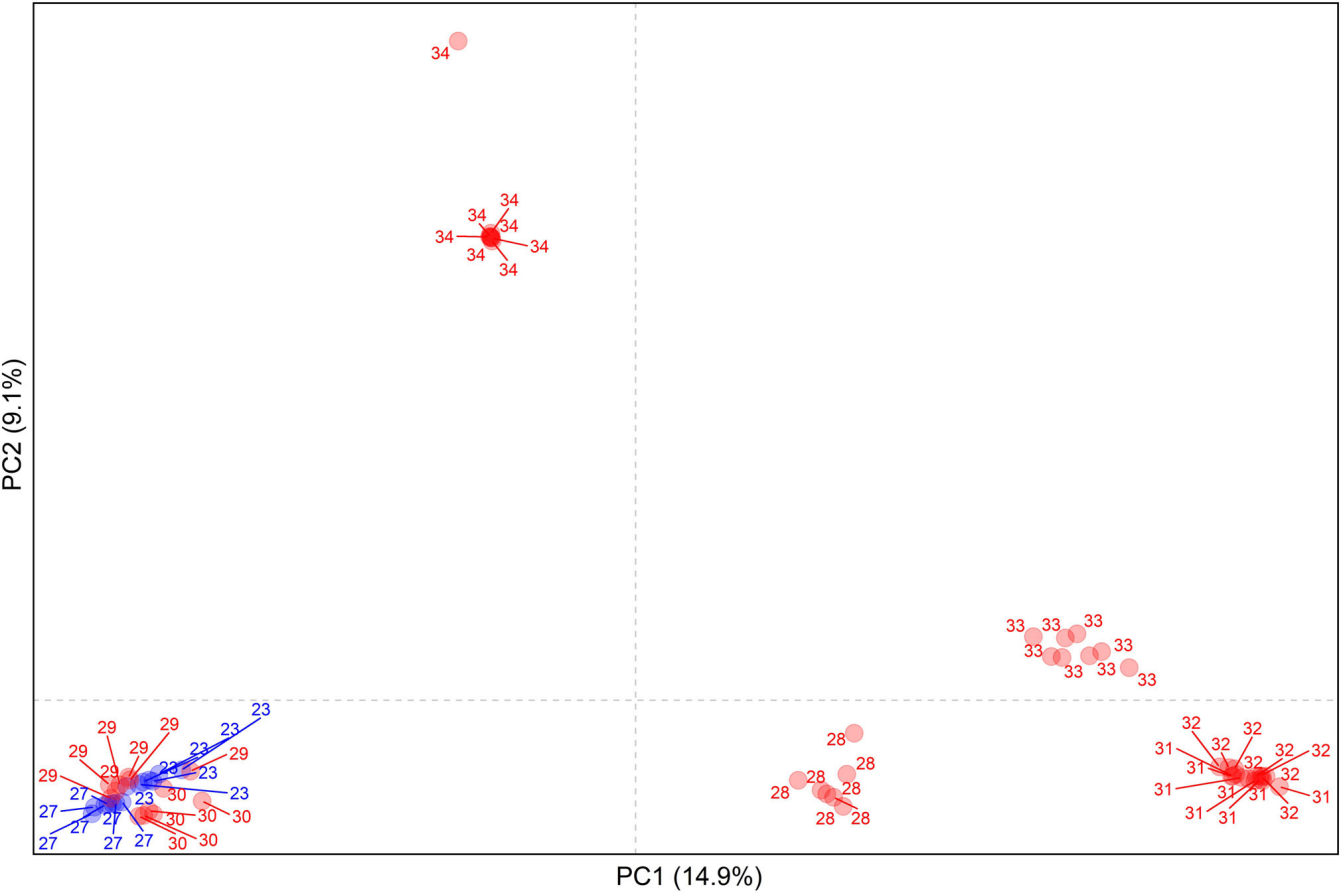
Principal component analysis (PCA) of *Amaranthus hybridus* populations. Labels in red and blue correspond to ALS‐inhibitor‐resistant and ‐susceptible populations, respectively.

Principal component analysis revealed clear genetic differentiation among *A. hybridus* populations (Fig. 2). Together, PC1 and PC2 explained 24% of the variance in the dataset and distinctly separated the populations. Populations from Mato Grosso (23, 27, 29, and 30) formed a distinct cluster, reflecting their geographic proximity. Notably, resistant populations 29 and 30, carrying the Asp‐376‐Glu mutation, grouped with susceptible populations 23 and 27, regardless of their resistance status, showing that geographic proximity overrides resistance‐related point mutations in the *ALS* gene. Population 34 from Goiás was distinctly separated from all other populations along PC2 (9.1%), indicating a greater genetic distance from the rest of the populations. Population 28 from Minas Gerais and 33 from Paraná were distributed along similar region of PC1 (14.9%), despite 33 carrying the Asp‐376‐Glu mutation and 28 carrying the Trp‐574‐Leu mutation. Similarly, populations 28 and 33 were positioned near populations from Rio Grande do Sul (31 and 32) along PC1 (14.9%), despite their geographical distance and differences in ALS mutations. This close clustering suggests a shared genetic background or potential gene flow among these populations. Additionally, populations from Rio Grande do Sul clustered together, despite 31 carrying the Trp‐574‐Leu mutation and 32 the Asp‐376‐Glu mutation. This indicates that independent evolutionary events may have occurred within similar genetic backgrounds, consistent with parallel evolution, where distinct resistance‐endowing mutations arise independently in closely related populations under similar selective pressures.

**Figure 3:**
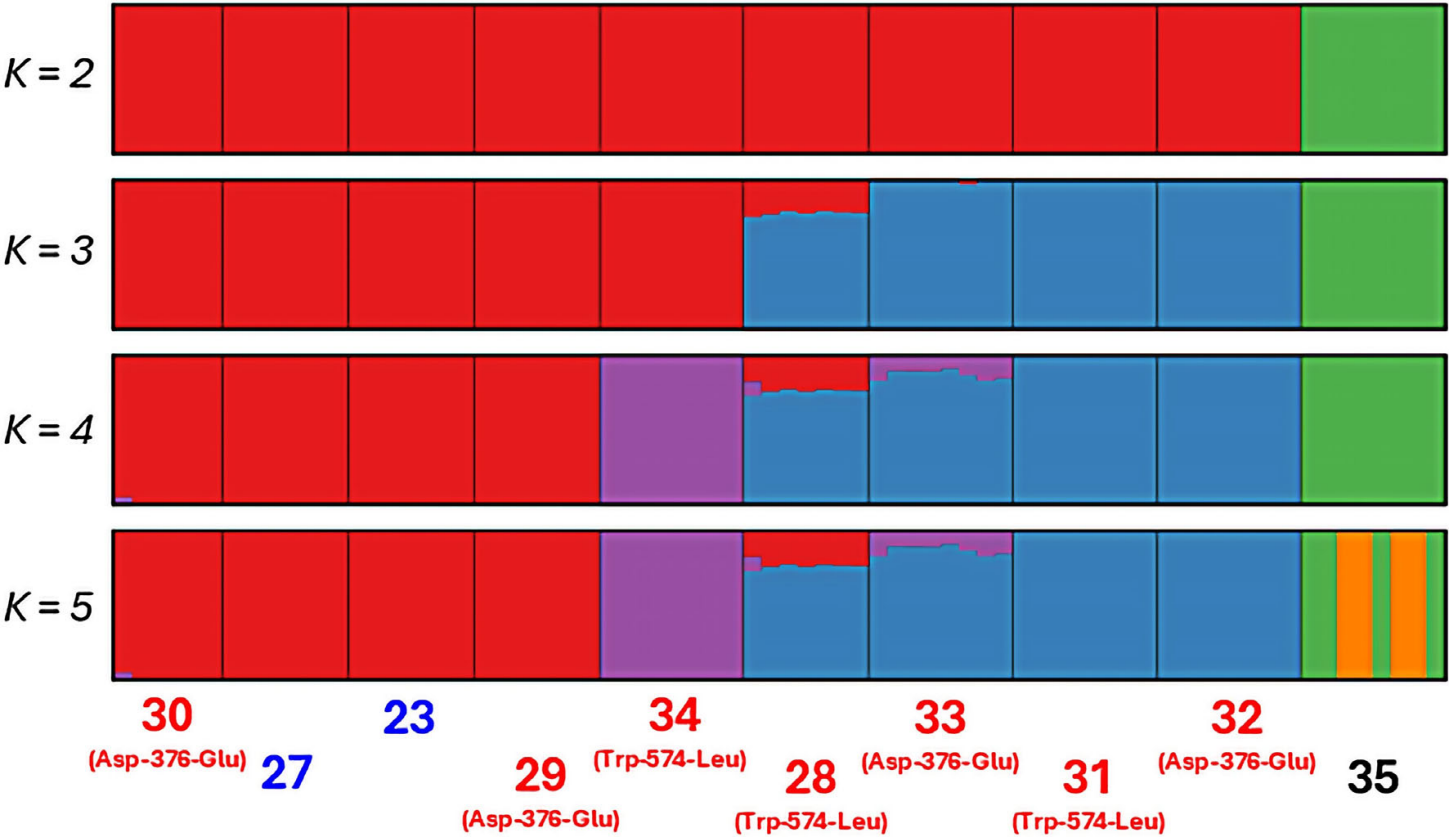
Admixture analysis of genetic clusters (*K*) in *Amaranthus hybridus* populations. Probabilities of assignment of individuals at *K* = 2 to 5. Labels in red and blue correspond to ALS‐inhibitor‐resistant and ‐susceptible populations, respectively. Population 35 is *Amaranthus palmeri* and was included as an outgroup.

ADMIXTURE analysis suggested distinct genetic separation between the *A. hybridus* populations (red) and the outgroup *A. palmeri* (35; green) with *K* = 2, where *K* represents the number of genetic clusters in the analysis (Fig. 3). As expected, the outgroup formed a unique genetic cluster, maintaining its distinction across all *K* values due to species‐level differences. As *K* values increased, additional genetic clusters emerged (purple and blue), revealing further structure within *A. hybridus* populations. A cross‐validation analysis (data not shown) indicated *K* = 5 as the most likely true value, where specific clusters corresponded to four particular groups of *A. hybridus* populations: 23, 27, 29, and 30 (red); 28, 31, 32 and 33 (predominantly blue); and 34 alone (purple) (Fig. 3). Evidence of gene flow was observed in 28 and 33 populations, indicated by mixed ancestries within individual populations. For example, all individuals within 28 contained both blue and red ancestries, with one individual possessing blue, red, and purple ancestries, suggesting ongoing hybridization between distinct genetic ancestries. In 33, all individuals contained both blue and purple ancestries. Overall, ADMIXTURE analysis results strongly corroborate with the PCA; however, most individuals in the analysis had a clear assignment to a single genetic ancestry.

**Figure 4:**
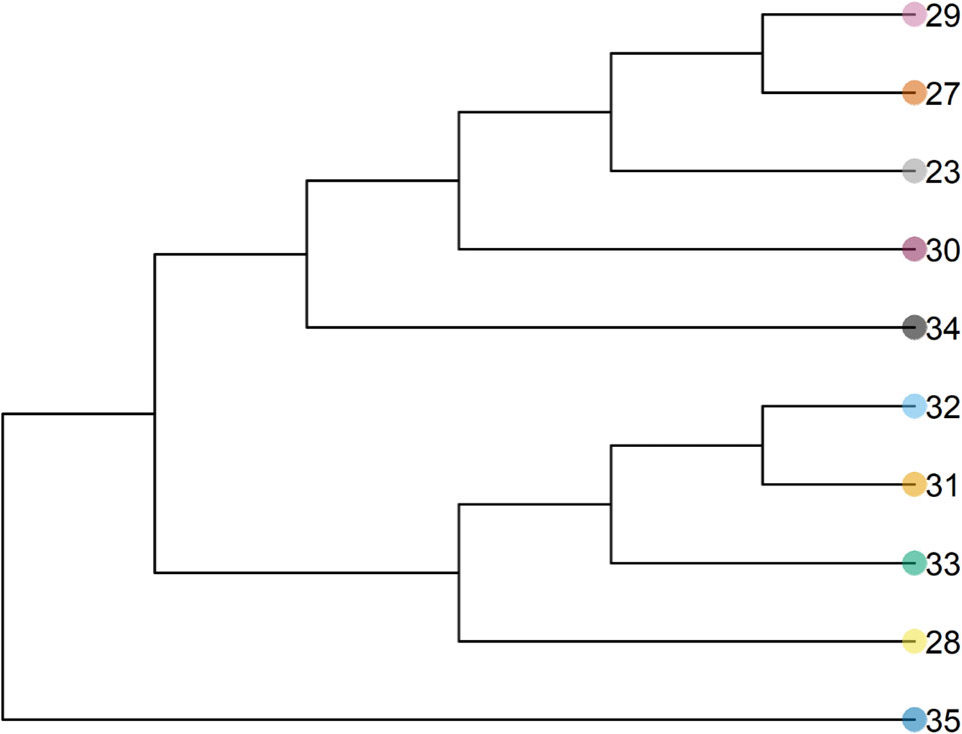
Rooted Neighbor‐joining tree including the *Amaranthus hybridus* populations and the outgroup (*Amaranthus palmeri*; 35).

**Figure 5:**
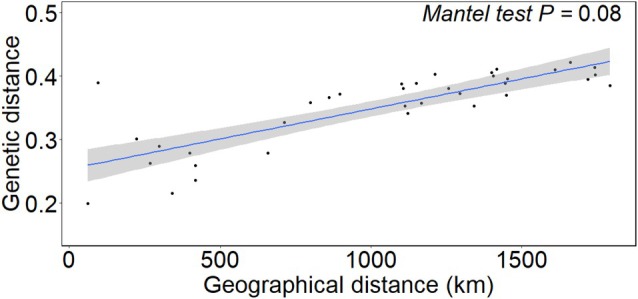
Isolation‐by‐distance plot with pairwise genetic and geographic distance between *Amaranthus hybridus* individuals. The mantel test was performed with 10 000 simulations (*P* = 0.08).

The Neighbor‐joining tree analysis supports the PCA and ADMIXTURE results (Fig. 4). For example, populations from Mato Grosso (23, 27, 29, and 30) and Goiás (34) appear in the top clade, whereas populations from Rio Grande do Sul (31 and 32), Paraná (33), and Minas Gerais (28) are clustered in the bottom clade. The Mantel test revealed a weak relationship between genetic distance and geographic distance (*P* = 0.08; Fig. 5). The linear regression between genetic and geographic distances had a positive, non‐zero *β* = 9.4 × 10^−5^. These results suggested that populations that are geographically further apart tend to be more differentiated, although the isolation‐by‐distance was weak in these analyses.

## 4 DISCUSSION

*Amaranthus hybridus* is a weed species of growing concern in South American grain production, and the evolution of herbicide‐resistant populations further complicates its management. Our results indicated that ALS resistance in *A. hybridus* populations was associated with target‐site‐based mechanisms. Two distinct mutations in the *ALS* gene were identified, both conferring resistance to chlorimuron. The presence of populations with the same amino acid substitution in different genetic clusters (as revealed by PCA, ADMIXTURE, and the phylogenetic tree) strongly supports the hypothesis of multiple evolutionary origins of ALS‐inhibitor resistance. Our ADMIXTURE data also suggest that gene flow could contribute to the spread of ALS‐resistant *A. hybridus* populations, although to a much lesser extent than independent evolution in the populations we studied.

Analysis of the *ALS* sequences revealed a single nucleotide transversion of TGG to TTG at codon 574, resulting in the Trp‐574‐Leu mutation in populations 28, 31, and 34. In contrast, populations 29, 30, 32, and 33 exhibited a substitution of GAT to GAG at codon 376, resulting in the Asp‐376‐Glu mutation. These findings underscore the role of TSR in the survival of *A. hybridus* populations treated with the ALS inhibitor chlorimuron. Our previous research indicated that resistant populations had GR_50_ values ranging from 37 to 100 g ai ha^−1^, while susceptible populations were controlled with 20 g ai ha^−1^ (labeled rate).^15^ Three populations previously reported as ALS inhibitor‐resistant in southern Brazil also exhibited variable levels of resistance to chlorimuron, with GR_50_ values of 27, 44, and 63 g ai ha^−1^.^6^, ^17^ Previous studies in *A. palmeri* have shown that *ALS* with a mutation at the Trp‐574 position is less sensitive to ALS inhibitors than at the Asp‐376 position.^45^ Interestingly, we could not establish correlations between the GR_50_ values we previously obtained, and the type of mutation identified in this research. For instance, population 31 had the Trp‐574 mutation and a GR_50_ of 52 g ai ha^−1^, while populations 29 and 33 had the Asp‐376 mutation with GR_50_ values of 40 and 100 g ai ha^−1^, respectively.^15^

Populations exhibiting the Asp‐376‐Glu mutation appear in different ancestries in the ADMIXTURE analysis (29 and 30 in the red ancestry, 32 in the blue), suggesting the mutations occurred in different genetic backgrounds. Similarly, populations with the Trp‐574‐Leu mutation appeared in the purple and blue genetic backgrounds. Taken together, these results provide strong evidence that ALS‐inhibitor resistance evolved independently, rather than a single evolutionary event followed by long‐distance dispersal. Similar mutations have been documented in *A. hybridus* populations from Argentina, which displayed cross‐resistance to both the sulfonylurea herbicide chlorimuron and the imidazolinone herbicide imazethapyr.^5^ Previous studies have further demonstrated the Trp‐574‐Leu and Asp‐376‐Glu mutations confer broad‐spectrum resistance across all ALS‐inhibiting chemical families, including imidazolinones, pyrimidinyl thiobenzoates, sulfonylureas, triazolopyrimidines, and triazolinones.^46^, ^47^, ^48^ In southern Brazil, two ALS‐resistant *A. hybridus* populations were identified carrying the Trp‐574‐Leu mutation, exhibiting cross‐resistance to the sulfonylurea herbicide chlorimuron (GR_50_ ≥ 26 g ai ha^−1^) and the imidazolinone herbicide imazethapyr (GR_50_ ≥ 94 g ai ha^−1^).^17^ These populations were also glyphosate‐resistant, carrying the TAP‐IVS mutation. Similarly, in Argentina, multiple independent mutations have been reported in an *A. hybridus* population resistant to glyphosate and ALS inhibitors, attributed to the triple amino acid substitution (TAP‐IVS) in the *EPSPS* gene and the Ser‐653‐Asn substitution in the *ALS* gene.^49^ The evolution of cross‐ and multiple‐resistant populations further complicates weed management in soybeans by limiting the availability of effective herbicide options in this system.

The population genomics statistics indicated relatively low genetic diversity among *A. hybridus* populations, with H_E_ ranging from 0.00117 to 0.01033 and π ranging from 0.00126 to 0.01119. Population 34, from Goiás state, exhibited the lowest H_E_ (0.00117) and π (0.00126), whereas population 30, from Mato Grosso state, showed the highest H_E_ (0.01033) and π (0.01119). These low levels of genetic diversity are consistent with the reproductive biology of *A. hybridus*, a monoecious, primarily self‐pollinated species. Self‐pollination limits gene flow and promotes homozygosity, reducing genetic variation within populations. Additionally, strong herbicide selection pressure from ALS inhibitors likely contributes to the loss of genetic diversity, as resistant individuals with specific mutations survive and reproduce, leading to a more homogenous gene pool while reducing other genetic variants. Variations in genetic diversity across populations may also reflect regional differences in environmental conditions, as well as the intensity of herbicide selection pressure and other management practices. These findings align with other studies that report low genetic diversity in self‐pollinated species, such as *Conyza bonariensis* (*H*_E_ = 0.00 to 0.35),^50^ *C. canadensis* (*H*_E_ = 0.00 to 0.45),^51^ and *Echinochloa oryzoides* (*H*_E_ = 0.089 to 0.693).^52^ In contrast, higher H_E_ values have been reported for obligated outcrossing *Amaranthus* species, such as *A. tuberculatus* (*H*_E_ = 0.367 and 0.628 to 0.211)^53^ and *A. palmeri* (*H*_E_ = 0.163 to 0.211).^54^ Likewise, in the outcrossing species *Lolium multiflorum*, *H*_E_ values ranged from 0.065 to 0.153, while π values varied from 0.070 to 0.152, indicating a high number of polymorphic events.^55^ The F_IS_ estimates, ranging from −0.0015 to 0.02997, indicated low to moderate levels of inbreeding among *A. hybridus* populations. Most F_IS_ values were close to zero, suggesting limited inbreeding, with some populations showing slight heterozygote excess or deficiency. Negative *F*_IS_ values suggest occasional outcrossing, which could indicate gene flow between populations, while positive F_IS_ values indicate moderate inbreeding, possibly reflecting a higher degree of self‐pollination or limited gene flow. These variations in F_IS_ may also be influenced by local environmental conditions and selective pressures, which could affect the balance between outcrossing and inbreeding across populations. Despite being an outcrossing species, the outgroup population *A. palmeri* exhibited a higher inbreeding coefficient (*F*_IS_ = 0.17421) compared to the *A. hybridus* populations. This higher *F*_IS_ might have been due to recent demographic events such as population bottlenecks, assortative mating, or reduced effective population size. Similarly, high inbreeding values (*F*_IS_ = 0.374 to 0.475) have been reported for glyphosate‐resistant *L. multiflorum* populations in California.^55^ These results were attributed to population bottlenecks, as indicated by heterozygote excess relative to expectations under a drift‐mutation equilibrium model.

Principal component analysis suggests that ALS resistance in *A. hybridus* evolved independently multiple times. The lack of a common genetic clustering among resistant populations with different ALS mutations supports the hypothesis that independent evolutionary events occurred in response to similar selective pressures across different populations. The clustering of populations primarily by geographic origin rather than mutation type implies that regional factors, such as local selection pressures, play a significant role in shaping genetic variation. This structure pattern supports the idea that resistance likely emerged independently within each region rather than spreading from a single origin. Similar results were observed in *L. multiflorum*, which evolved resistance to acetyl‐CoA carboxylase (ACCase) inhibitors through multiple independent evolutionary events involving different mutations at positions 1781, 2027, 2041, and 2078 in the *ACCase* gene.^37^ Likewise, in Italy, the ALS mutation Trp‐574‐Leu evolved at least three times in geographically separated *A. tuberculatus* populations, indicating multiple evolutionary origins of resistance.^53^

ADMIXTURE analysis provided evidence of the genetic homogeneity within populations, with some admixture among populations. These findings align closely with the PCA results, which also revealed genetic clustering patterns corresponding to geographic locations. Specifically, populations from Mato Grosso state (23, 27, 29, and 30) were assigned to the red cluster, suggesting a shared genetic background distinct from other regions. Similarly, populations from the Rio Grande do Sul state (31 and 32) grouped into the blue cluster, while the Goiás population (34) formed an isolated purple cluster, suggesting a unique genetic composition. Notably, populations from Minas Gerais (28) and Paraná (33) exhibited mixed ancestries, suggesting gene flow or historical hybridization among clusters. Population 28 from Minas Gerais showed blue, red, and purple ancestries, suggesting that this region may serve as a convergence point for gene flow among populations from Rio Grande do Sul, Mato Grosso, and Goiás states. Similarly, population 33 from Paraná displayed both blue and purple ancestries, suggesting admixture between the Rio Grande do Sul and Goiás populations. These results are consistent with findings from other ADMIXTURE analyses on herbicide‐resistant weed populations. For example, glyphosate‐resistant *A. palmeri* populations from different geographic regions of the United States exhibited low to minimal admixture of resistance alleles, suggesting two possible scenarios: independent origins of glyphosate resistance or migration events *via* seed movement.^54^ Similarly, low levels of admixture were detected in ALS‐resistant *A. tuberculatus* collected from soybean fields in Italy.^53^ Gene flow involves the movement of genetic material (alleles) between populations of the same or different species, typically driven by migration, reproduction, or hybridization.^56^ This exchange introduces new genetic variations into a population, potentially altering its genetic structure and influencing its evolutionary trajectory.^57^ Overall, we observed limited variability within populations, as evidenced by the ADMIXTURE analysis, where individuals within a population had similar assignments, and the PCA, where individuals within a population clustered together. Population genomics summary statistics further support these observations due to the low standard errors around the mean values.

The isolation‐by‐distance analysis indicated weak isolation among populations. In other words, populations tend to be more genetically distant as geographic distance increases. These results are not surprising, considering that the PCA and ADMIXTURE analyses suggest independent evolutionary events have contributed to the evolution of ALS inhibitor resistance. The isolation‐by‐distance findings are further supported by the phylogenetic tree, as populations with distinct resistance‐endowing mutations (*i.e*., Asp‐376 or Trp‐574) do not necessarily fall within the same clade. For example, population 32, which has the Asp‐376 mutation, is closely related to population 31, which has the Trp‐574 mutation. These results further suggest that resistance‐endowing mutations arose independently in similar genetic backgrounds. Similar findings were observed in ALS‐resistant *Lactuca serriola* populations in Australia, where four distinct ALS mutations (Pro‐197‐Thr, Pro‐197‐His, Pro‐197‐Ser, and Pro‐197‐Leu) were identified across resistant populations.^58^ Genetic relationships among populations, based on IRSS marker variation, indicated that resistance spread through both independent selection events and seed dispersal.^58^ A Mantel test in the study revealed no significant correlation between genetic and geographic distances, suggesting that the distribution of populations was erratic and not influenced by geographic proximity.^58^

In *Amaranthus* species, gene flow can occur through the dispersal of pollen or seeds.^59^, ^60^ Weed seed dispersal is often more effective than pollen in spreading herbicide‐resistant alleles over large distances.^61^ The observed long‐distance gene flow among *A. hybridus* populations across different soybean‐growing regions in this study may be associated with human activities, particularly the movement of agricultural machinery. Soybean planting generally begins earlier in Brazil's southern regions due to distinct precipitation patterns. This timing difference allows equipment used in the south to be shared with northern regions once operations in the south are completed. The exchange of agricultural machinery is facilitated by farmers who own land in both regions and by third‐party companies offering custom farming services in multiple locations. A previous study provided evidence of gene flow of glyphosate‐resistant *Digitaria insularis* across different soybean‐growing regions in Brazil, suggesting that the movement of the species was associated with agricultural machinery, particularly in a south‐to‐north direction.^31^ It is also possible that the trade of non‐certified soybean seed lots contaminated with *A. hybridus* seeds contributes to resistance dispersal.

Continued selection of ALS‐resistant *A. hybridus* populations is concerning because it limits effective herbicide options for controlling this species in soybean production systems. Therefore, managing herbicide‐resistant *A. hybridus* populations will require integrated weed management programs that combine chemical and non‐chemical approaches to manage existing resistance, reduce selection pressure for future resistance, and limit the spread of resistance through gene flow.

## 5 CONCLUSION

Acetolactate synthase resistance in *A. hybridus* evolved independently across soybean‐growing regions in Brazil. Resistance to chlorimuron is associated with the target‐site mutations Trp‐574‐Leu and Asp‐376‐Glu. *Amaranthus hybridus* populations are genetically distinct by geographic region, with two populations showing evidence of admixture. The spread of ALS resistance alleles is likely driven by local, independent evolution and, to a lesser extent, by gene flow through seed dispersal.

## CONFLICT OF INTEREST DECLARATION

No conflicts of interest have been declared.

## Data Availability

The data that support the findings of this study are available from the corresponding author upon reasonable request.
